# Northern and Southern Hemispheres

**Published:** 1834

**Authors:** James Lakey

**Affiliations:** Cincinnati


**Art. II.**—*Thoughts on the Moral and Intellectual Energies of Man in the Northern and Southern Hemispheres.* By Dr. James Lakey.

If a travelling philosopher from Jupiter or Saturn, a modern Micromegas, were to land upon our little earth and become acquainted with all its written languages, among the many novelties that would attract his attention, would be the amazing difference in mental energy between the men of the northern and southern hemisphere.

The fact of the superiority of the north over the south is admitted by all observing men, but, as yet, I believe no one has assigned any physical cause for this apparent phenomenon.

I assume an hypothesis,—and give the following as two of the principal causes.

1. The greater obliquity of the sun's rays in the southern hemisphere.

2. The immense surface of ocean lying in the southern hemisphere.

The man of the north enjoys about eight days more of the annual sun than his brother of the south, which, allowing 6000 years for the age of the world, makes an aggregate of 131 years for 180 generations of men. Every astronomer knows that the sun passes sooner through the southern signs than through the northern;—that is, that the sun travels from the equator to Capricorn and from thence back to the equator in a shorter space of time than from the equator to Cancer, and from Cancer back to the equator. This is caused by the earth's place in her orbit being nearer the sun in winter than in summer by about 3,000,000 of miles;—the earth being in her *perihelion* in December and in her *aphellion* in June. She of course would move swifter in her orbit as she approached the immense body of the sun. The sun himself not being exactly in the centre, but in the lower *foci* of the earth's orbit, adds to this inequality, and gives more of his vertical rays to the north than to the south.

It is a familiar truth in philosophy that a cause, however small, steadily and incessantly operating for ages, must produce stupendous effects. These causes having existed ever since creation, let us look at their effects upon the men and animals that inhabit the respective sides of the equator.

I propose to establish the following facts.

1. That about five sixths of the land on the globe lies in the northern hemisphere.

2. That this land is generally far superior in fertility and productiveness.

3. That the men and land animals are superior in physical, moral, and intellectual energy.

The northern hemisphere embraces the whole of North America and about one fifth of South America. The equatorial line cuts South America from the city of Quito on the west, to the mouth of the majestic Amazon on the east. The northern hemisphere takes in the whole of Europe and Asia from Borneo to Behring's Straits. It also includes about three fourths of the African peninsula, extending southerly from Gibraltar to the Gulf of Guinea. From thence the equator, leaving the island of St. Thomas on the north, extends east to the Indian Ocean, cutting the northern section of the barbarous province of Zanguebar. The amount of land contained in the southern hemisphere, compared with that of the northern, is very small. It contains the southern section of South America, South Africa, New Holland, New Zealand, Papua, part of Polynesia, and a few islands.

The southern hemisphere is much colder than the northern. Cape Horn and Sandwich Land lying between 54 and 58 degrees of south latitude, are rendered totally uninhabitable to civilized men, by the intensity of the cold. Edinburgh, Copenhagen, Moscow, Stockholm and St. Petersburgh, lying in equally high latitudes north .are the crowded seats of civilization, science and commerce. Modern geographers tell us that in latitude 48 south, the *summer temperature* of the air is not warmer than the *winter temperature* of 42 north;—in other words, that the summer of Patagonia is no warmer than the winter of Boston, Italy and Spain.

The heat of the warmest months (December and January) in Patagonia is not more than 45, 5 while at Petersburgh, lying 11 degrees nearer to the Pole it is 66!!!

Snow falls almost every day in South America in latitude 52 and 53, and the thermometer rarely rises above 52 degrees. Sir Joseph Banks lost two of his attendants from extreme cold in this region during a night in midsummer. Human habitations are found as high as 78 north—but none higher than 54 south.

How can this immense disparity be explained? Will the eight days' annual absence of the sun, added to the wide waste of water in the southern hemisphere cause this great diminution of heat? I know of no other causes—and these having been in constant operation for ages, have established the superiority of North over South America. The superiority of North to South Africa from the same causes is equally unquestionable. Hence the second position is established without difficulty.

I now come to the third position which has been laid down as an hypothesis,—not doubting but at some future period, abler pens and more thorough research will sustain both the theory and the facts. I assert, then, that the man of the south is, and ever has been, inferior in mental energy to the man of the north; and that this inferiority is chiefly owing to climate. That climate, even in the same latitude, and in the same hemisphere, exercises great influence upon men will not be denied. What but climate could create the amazing difference between the Hollander and the Frenchmen, the Irish and English, &c.? They all belong to the Caucasian race of men. Let us examine this subject in detail.

The Spaniards at the commencement of the 16th century found the Mexicans further advanced in the arts than the Peruvians. Cortez had fiercer foes to encounter and conquer than were those who fell beneath the sword of the illiterate Pizariro. Three hundred years have elapsed, and the contraststill remains. The mere politician who never troubles his head with theories, tells us, that Mexico will take her rank among the nations of the earth sooner, and that her station will be higher than that of Peru, La Plata or Chili. Let it be remembered that these Hispanio-Americans descended from the same ancestors; and that these ancestors were emigrants from one of the richest, most renowned and warlike of the European nations. The modern Spaniards, according to Dr. Morse, have more of the ancient Roman blood in their veins than any other existing nation. Mexico and Colombia have produced Iturbide and Bolivar,—but where are, or ever were, the distinguished men born south of the equatorial line?

Of the African peninsula we observe that in the northeast lies ancient Egypt, the parent of civilization and science—on the north lie the Barbary states, degenerated it is true, but even Morocco, Tunis and Tripoli are infinitely superior to any South African nation. North Africa has produced a Hannibal, a Jugurtha, a Syphax, a Cyprian, and a host of distinguished characters. Ancient Ethiopia, now called Nubia and Abyssinia, must have been a state of great wealth and importance. Sacred history informs us of Zerah, the Ethiopian, who invaded Palestine with 1,000,000 of soldiers. Owing to the want of figures in the early ages—the carelessness of copyists, and the credulity of commentators, this incredible number still remains in our Bibles. The mistake in numbers does not, however, invalidate the fact or weaken the argument. Every one believes that Xerxes invaded Greece—but none but school-boys believe that he brought with him 5,000,000—3,000,000, or even 1,700,000 men. The Ethiopian general, some seven or eight hundred years before Christ, invaded Judea with an army of, no doubt, 100,000 combatants. He must have marched an immense distance, and nothing but a certain degree of civilization and industry could have supported such an army. The same resistless spirit of conquest that led this black Alexander and his warlike Ethiopians to Asia, would have led them to South Africa, had there been any thing there to invite an invader.

At a later period the powerful republic of Carthage ruled the Mediterranean. Her merchants, seamen, and soldiers were in some respects to the ancient world what those of Great Britain are to the modern. She planted colonies in Cadiz and Cornwall, and sat like a queen among the nations.

But what of South Africa? History has not recorded nor romance feigned the existence of any authors, heroes or statesmen in South Africa. The North Africans have degenerated—the South Africans have not, for they have ever been savages. I speak of the native population. No ruins of ancient cities show that civilization ever existed south of the line.

The descendants of the Dutch, French and English unquestionably degenerate at the Cape of Good Hope. The lion of the Cape is less fierce and courageous than the lion of the Lybian and Sahara desarts. The same is said to be true of other animals. The Romans and Carthagenians obtained their lions and elephants wholly from the north. The North Africans were expert horsemen in early times—the South Africans were not, and are not. The North African "trained him to his hand the embattled elephant laden with war." The South African never has tamed the elephant even for domestic purposes! In short, it may be asked where is a single civilized nation that exists, or ever did exist, in this peninsula, south of the line? Has the entire southern hemisphere, from the equator to the south pole, produced one distinguished general, one author, poet, or legislator? I think not—or if any one has been accidentally born there, he must have been the issue of northern parents.

Will it be said that the southern hemisphere was settled later than the northern? Admitted; but 300 years have elapsed since the Spaniards and Portuguese planted themselves in Paraguay and Brazil—and are not three centuries sufficiently long to test the principle? The aboriginal inhabitants of this hemisphere are inferior to those of the north.

The natives of New Holland are the most degraded, debased, and filthy savages on the face of the earth.

The New Hollanders, although living between 10 and 40 degrees of south latitude, fall far below in point of intellect the Esquimaux, the Greenlanders, and the Laplanders. Do not the descendants of the Europeans degenerate in the southern hemisphere? I think that the lapse of a few ages will add new proofs to this hitherto unexplained fact;—and that the continent (or island) of New Holland, even if peopled by Englishmen, will never rival or even equal in importance the Canadas.

The aborigines inhabitants of South Africa have stubbornly resisted all efforts directed to their civilization. The filthy and abominable habits of the Hottentots are well known. An untanned sheepskin serves him for a garment in summer and winter, and for a winding sheet when he dies. Let us look at these South Africans closer. Their country has no doubt been settled at least 3000 years, and they could have had access by land to Egypt and from thence to Arabia, the two cradles which once contained all the science of the the infant world. They must have traversed the torrid zone, but a few degrees of travel would have brought them to the head waters of the Nile, from whence they could have floated down upon the southern shores of the Mediterranean. Our savage ancestors, the barbarians of the north, travelled a much greater distance by land in order to overturn and ravage the Roman empire. The North American Indian would have made a small thing of such a journey to have gratified his thirst for war and revenge.

I have said that the descendants of the Dutch have strangely degenerated at the Cape, so say the English writers, and it is no doubt true. The English themselves may soon degenerate and change, as their countryman John Newton said, from WHITE men to BLACK, not in color but in disposition.—They conquered the Cape in 1806, but a little over one quarter of a century is not long enough to shew a great degree of degeneracy. The English population at present bears no proportion to the numbers of Belgo-Africans who surround it. If the Dutch have degenerated at the Cape, why have not the descendants of the same nation degenerated at Eustatia, at New York and New Jersey?

Are the people of the southern hemisphere destined to be forever a prey to ignorance and barbarism? The Christian philanthropist will say "no!" They may advance, but reasoning from what has been, and now is, their position will ever keep them at an immeasurable distance behind their brethren of the north.

Let the isles of New Zealand be compared, not with Spain or Sardinia, which lie at an equal distance from the line—nor with Great Britain, which resembles them in climate, but with Iceland. In every thing depending on human exertion or human virtue, the Icelander is superior to the New Zealander.

A good history of Brazil, written by a native of that country, is, I believe, a desideratum in the republic of letters.—There are, no doubt, many of the descendants of the Dutch in that region. If that vast country, equal in extent to the United States, has ever produced any eminent men in the arts of war or peace, the fact is not generally known. It will be said that Portugal herself has degenerated. I know this, but it was not during the period of her degeneracy that she conquered and colonized the immense territory of Brazil. She was then in the vigor of manhood, her sailors, mathematicians and merchants stood among the foremost of Europe.

Will it be said that these remarks apply with equal force to the entire torrid zone? I think they do not; but am willing to admit that the shades of difference are not so apparent between the southern and northern sections of the torrid zone, as between the southern temperate zone and the northern temperate zone.

Let us, then, leave the burning zone, which the ancients erroneously asserted to be uninhabitable and impassable, and which all the moderns admit to be unfriendly and depressing to intellectual activity.

Before speaking of the north and south temperate zones, let us take a short view of the Polar circles. The Arctic circle is said to contain 2,520,000 square miles of land, which includes the greater part of Greenland, the north end of the American continent, and the entire northern boundary of the Russian Empire.

Most of this land is uninhabited and uninhabitable. It furnishes, however, some valuable articles for commerce, and the Greenlanders and Laplanders are said to be industrious, happy and contented.

What has the Antarctic Circle compared with this? Nothing but ice and an open sea. Geographers tell us of 200,000 square miles of land in the southern circle, but have said nothing of its latitude, and we may search in vain for any inhabited or inhabitable land. The antarctic circle must be composed nearly or wholly of water.

The temperate zones, then, are the true abode of civilized men. A bare comparison between the northern and southern temperate zones would seem sufficient to establish the immeasurable superiority of the former in every respect.—The northern zone contains much the most land.* It embo-

---

*According to the best geographers, the superficies of the earth contain 190,992,000 square miles; of which the water surface is 160,152,000, and land surface, or area, 38,840,000, or not quite one fourth part of the whole superficies.

Table I.—Summary of Oceanic Area, Southern, Indian and Pacific Oceans,

| | |
|---|---|
| Southern, Indian and Pacific Oceans, - - - - - | 126,045,000 |
| Atlantic, Mediterranean, Black, Baltic, Hudson's Bay and Arctic Oceans, - - - - | 33,857,000 |
| Caspian Sea, Lakes in America and other smaller collections of water, - - - - | 250,000 |
| Area of water, - - - | 160,152,000 |

Table II.—Land Area.

| | |
|---|---|
| Polynesia, or Oceanica, - - - - - | 100,000 |
| Austral Asia, - - - - - - - | 3,000,000 |
| Asia, - - - - - - | 11,500,000 |
| Europe, - - - - - - | 3,020,000 |
| Africa, - - - - - - | 8,000,000 |
| America (including Greenland,) - - - | 13,220,000 |
| Amount of Land area, - - - - | 38,840,000 |

dies most of the population, knowledge, and wealth of our little world. Let us take a bird's eye view of the northern zone, and first on our own continent. It commences about the middle of the Mexican Gulf; the tropic of Cancer crosses the republic of Mexico and cuts the southern end of the Californian peninsula. Between this and the Arctic circle, as the continent grows broader, lies an immense tract of land including four-fifths of Spanish North America, the United States, and all the continental parts of British, Danish, and Russian America. This tract contains an area of more land than the entire southern zone.

Little more than 300 years have elapsed since white men planted themselves upon the soil of North America, and now she exceeds in population, property, and in acres of cultivated land, the entire temperate zone of the southern hemisphere.

Our own republic possesses and exercises more political, religious and moral influence, and she has been settled but about two centuries.

At present I shall leave Europe, and Asia wholly out of the question, well aware that those countries have been called the cradles of the human race, the store-house and nursery of nations, &c. and that the over-whelming superiority of these regions over the southern zone has been at-

Table III.—Land in the several Zones.

| | |
|---|---|
| Torrid Zone, | 13,600,000 |
| Southern Temperate Zone, | 3,560,000 |
| Northern Temperate Zone, | 17,960,000 |
| Southern Polar Circle, | 200,000 |
| Northern Polar Circle, | 2,500,000 |
| Total, | 28,840,000 |

Table IV.—Population of the earth.—(According to M. Adrian Babbi.)

| | Population. | Square Miles. |
|---|---|---|
| Europe, | 227.700.000 | 2.793,000 |
| Asia, | 390.000.000 | 12,118,000 |
| Africa, | 60.000.000 | 8,516.000 |
| America, | 39.000.000 | 11,046.000 |
| Oceanica, | 20.000,000 | 3,100.000 |
| Total, | 727,000,000 | 37,573,000 |

tributed to moral rather than physical causes, such as longer settlements, &c. &c. &c. &c.

It would be absurd to deny the immense influence of moral causes. But too much has been attributed to them. If the descendants of intelligent Europeans degenerate when transplanted to the south of Capricorn, and if this degeneracy is constant and uniform from generation to generation, it must be owing not to moral, but to physical causes.

Empires have arisen and fallen in Europe and Asia.—Whole nations have degenerated and become extinct.

> ——————" * * Empires die!
> Where now the Roman? Greek?
> They stalk an empty name,
> * * * * * * * *
> Though half our learning is their epitaph."

This can be proved by ruins as well as written records.—But the people of the southern zone have never degenerated! and why? They were never civilized. They had nothing to degenerate from. No writ of ejectment could reach or affect a houseless pauper. Having had no grandeur, they have had no decadence.

Let us look upon the southern zone, upon our own continent—evidently the best part of that zone. The tropic of Capricon cuts from Rio Janerio on the east to the northern end of Chili on the west,---leaving the greater part of Buenos Ayres, a small corner of Brazil, all Chili and Patagonia, together with some unimportant islands to the south, which go to make up the South American temperate zone.

Now these countries have been longer settled than the United States, and what is their present situation? Low enough, as far as intellect is concerned. The soil of Buenos Ayres is little cultivated, and the intellect of the people still less. The natives of this province have as yet manifested but a small degree of that intelligence, ardent energy, and enterprise so necessary for a young nation. Most of the officers that fought the battles of their revolution were foreigners. The people are well described in the following extract from Flint's Geography:

"It has been computed that the shepherds of these plains [La Plata] tend 12,000,000 of oxen. But in this delicious climate, and on this luxuriant soil, the people degenerate to DEMI-SAVAGES, and are ignorant, indolent, and miserable. They live in mud cottages, and gaming is their predominant passion." Vol. ii. p. 163.

Before their revolt, the Buenos-Ayreans had colleges but no printing presses

It is customary to account for the debasement of these people by attributing the cause *wholly* to the despotism of the government, and the tyranny of the priests. This will not do.—Learned men flourished under the rule of Louis 14, and Cervantes and Quevedo wrote and lived under the iron despotism of old Spain. The absolute monarchy of Prussia has myriads of learned men. Spain herself was never free—yet she had literature.

Patagonia is abandoned to desolation and barbarism. Terra del Fuego is in the same latitude with Edinburgh. South Georgia, South Shetland and Sandwich land, all lie in the southern temperate zone,—but they are bleak and barren rocks, inhabited only by sea-birds and seals.

The northern zone of Africa includes all the native science that exists in that peninsula. The southern zone embraces a territory of about 11 degrees in length, embracing Caffraria, and Cape Colony. This last has been in possession of highly civilized christian protestant nations for nearly two centuries. The Dutch planted the proud standard of their republic at Cape Town in 1652. It was the asylum of French protestants who fled from religious persecution. The English succeeded the Dutch in 1806. The colony is said to contain 12,000 white inhabitants, evidently in a state of degeneracy. Here it cannot be said with truth as has been asserted of Buenos Ayres and Brazil, that the people of the parent states have degenerated.

France, England and Holland stand higher in civilization and science than they did in the 17th century. The historian of the 20th century will be able to discern the difference between the descendants of Englishmen at the Cape and the posterity of Frenchmen in Algiers.

The southern zone includes rather more than half of New-Holland and the whole of Van Dieman's land. The importance of these countries to the British crown is undoubtedly great;—but whether they are ever to be the abode of a dense and civilized people, is an unsettled question. If the theory which I have endeavoured to defend by collecting and arranging facts be correct, these countries can never rise to importance. Centuries will roll away and the descendants of Englishmen will continue to be ruled by a remote island. The Anglo-Australian will fear the rod of a master 15,000 miles off.

Much has been said of the exuberant fertility of Van Dieman's land. These accounts are to be received with caution. The natives of this island are among the most degraded of the human race. They are ignorant of agriculture, of the services to be derived from the use of animals, and of the use of metals. If the soil be fertile, they derive little or no sustenance from it. Caterpillars, serpents and spiders enter largely into an Australian bill of fare.

If at some future period some powerful nation should arise in the southern zone and become to that region what Great Britain and France have been, and now are to the northern zone,—why then this theory must be abandoned as erroneous, and these speculations be buried with other rubbish. It matters not whether this powerful nation, *that is to be*, be Anglo-Australian, Hispanio-American, Lusitanio-American, or Anglo-Belgo-African. If on the contrary the northern zone should continue to hold her immeasurable and unquestionable preponderance, the fact of the inferiority of the south will be too firmly established to be shaken.

The degeneracy of man in every degree of longitude in the southern zone is too uniform and general to be the result of accident or moral causes. The effect of physical causes will be understood and asserted by geographers and statesmen—and have weight with all commercial nations in planting colonies. Time will show the kind of men that are to inhabit Australia.

Dr. Good says: "the natives of New South Wales have no aptitude and learn nothing. The actual possession of the country by a British public and a British government, with a perpetual intercourse and the kindest encouragement, has made little or no impression upon the natives."

Dr. Caldwell says: "New South Wales extends beyond the 30th degree of south latitude, and has in many large sections a climate peculiarly delightful and healthy. Yet the following is the description of its inhabitants, given by Malte Brun. "New South Wales seems to offer their *native* varieties of inhabitants *all* belonging to the race of Oceanian negroes. In the neighborhood of Glasshouse Bay the savages have large heads, which in shape resemble the Ourang Outang. Their very limited intellects, their hairy bodies, and habitual agility in climbing trees, seems to bring them near to the monkey character. * * * In many other parts of the Oceanian continent, where the climate is by no means intensely hot, the people are of the same degraded character. They have woolly hair, set in tufts, a skin of a soiled and faded black, narrow, low retreating foreheads, flat noses, thick lips, a projecting muzzle, large and strong maxillary bones and teeth, and a defective chin, or rather no chin at all, their mouth being placed almost at the bottom of the face. Their stature is short, and their intellectual endowments extremely limited. They are in all respects inferior to the negroes of Africa."

These, then, are the New Hollanders—and here is one of the largest spots of arable earth in the southern zone. Where can we find the counterpart of this humbling description? No where between the tropic of Cancer and 40 north latitude. No where between 40 and the north pole. Such degraded and imbecile beings as these are not to be found even in the Arctic circle. How shall we account for this? Shall we adopt the learned Doctor's ingenious theory of man? Shall we suppose that the human race sprung from different original stocks? I shall not meddle with this recondite subject—the origin of man. I think we are to look to physical causes as having produced man as he is, and that the southern zone abounding in water and air is better calculated for birds and fish than for men.

The situation of New Zealand as it respects latitude (between 31 and 48 S.) is more favourable in the southern zone than is Great Britain in the north. Who are the New Zealanders? and what are their habits? They have shown themselves the most ferocious savages that the world has witnessed. Christian missionaries sent there for their conversion have been murdered and devoured,—literally eaten by these benighted cannibals.

"Attacked by intense cold, (says the Westminster Review, and buffetted by furious winds, these savages have gradually assimilated their character to that of their climate, and become rude, fierce, boisterous and unpitying. There country producing little or nothing during the winter months, they are forced during summer, when fish is abundant and easily caught upon their coasts, to smoke and dry vast quantities, which are laid up against inclement weather, as well as to provide against the chance of being besieged in their abodes by inimical tribes * * * * * they are cannibals from necessity. * * * * * Here, without the aid of "religious prejudices," we have the whole theory of cannibalism. Men, tortured by insufferable hunger, cast "wolfish eyes" upon each other, and by degrees conquering the strong repugnance which all animals feel to prey upon their own species, exactly from the same cause which impelled the African hyenas mentioned by Bruce, to eat their companions. * * * There is no nation, however, so openly and disgustingly addicted to anthropophagy as the New Zealanders. Their unnatural and ferocious appetite delights in the taste of a human victim. "In consequence of their abominable customs, (says M. Lessen,) these people have acquired a decided appetite for human flesh, and reckon among their *white* days those solemn festivals in which they can eat their fill of their favorite food. A chief of a New Zealand village confessed to the French officers that he experienced extraordinary gratification in devouring a corpse, and informed them that the brain was the most delicate bit, though the haunches were more substantial!! * * * * * The New Zealanders imagine that the spirits of their victorious fathers hover in the blast over their native villages, and then plunging into the glittering waves near the north Cape, repair to the Elysium prepared for them, &c. The souls of those on the contrary, who are slain in battle and devoured by their enemies, are eternally unhappy, and it is said to be for this reason, from a spirit of revenge that would do honor to the heart of a Grand Inquisitor that they are so anxious to feed upon their foes. They are desirous not only to have them dead but damned!"

It would be difficult to produce parallel instances of cannibalism in the temperate zone of the north, or even in the Arctic Circle. European Zealand, which gave the name to the South Sea Zealand, contained, in former ages, pirates of the fiercest kind;—but we have no record of cannibals. Great Britain has been peopled by pagans, by ferocious and warlike savages, but Cesar found no cannibals among the ancient inhabitants of that Island. The learned London Reviewers say, that the New Zealanders are cannibals from necessity. If so, with due deference let it be asked, does not the same, or even a greater degree of *necessity* exist within the limits of the Arctic Circle? Yet the Greenlanders, the Samoides, and the Esquimaux are not man-eaters.

I shall close this letter with a few more facts and arguments, without attending to the niceties of arrangement. Navigators have penetrated the Arctic Circle to within 10 or 12 degrees of the north pole, while few or no ships ever entered the Antartic Circle, and no land is laid down there. Men have braved the Arctic regions with impunity, and passed the long winters as high as 74 or 75. It is far from certain that animal life exists at all, even under the ice within the Antartic Circle.

Learning has flourished to some extent near the verge of the north frigid zone. Iceland and Lapland have had their learned men. Linnæus was reckoned among the *hyperbozean* learned, but whoever heard of the Australian learned? Whoever heard of the Polynesians learned, or of *any* learning between Capricorn and the south pole?

Great Britain alone, with her 24,000,000 of inhabitants, and her 100,000 square miles, (about half the size of New Zealand) possesses and exercises more moral and physical force than the whole southern zone, with her 4,000,000 of square miles of land, and her uncounted millions of semi-barbarians, upon whose territory the sun never sets.

The Arctic Circle has land animals of prodigious size and strength,—the Antarctic having no land, can have no animal life but what inhabits water. It may be doubted whether there is any land animal south of the tropic of Capricorn equal in size, strength and ferocity to the grizzly bear of the Rocky Mountains, or to his white brother of the Arctic Circle. The south may be superior to the north in the number and size of its ocean animals.

I have said that the hebitude and imbecility of intellect manifested by the people of the southern zone, was not wholly owing to the eight days' annual absence or obliquity of the sun; although that stands first in the list of causes. The observer in examining the southern zone is struck with the fact of its having "such wastes of ocean and such scanty land." It is compo ed almost wholly of water.

> "* * * Cynthia when to *southern* skies
> Spends her sweet beam upon the barren main."

Does not the immense mass of water have an unexplained, but deleterious effect upon the atmosphere, soil and men of these regions? Is not the latent heat of the earth less at the southern tropic in consequence of being surrounded by such vast bodies of water?

Future discoveries must settle these questions.

The earth was evidently not made for man and land animals alone. By far the greatest bulk and weight of animal life exists in water, either beneath the ocean or in fresh water.

Nine-tenths of the southern hemisphere being covered with water, the countless millions of ocean animals which inhabit there must far outnumber those who plough the great deep of the northern hemisphere.

In calling attention to this new theory, I have endeavored to be sparing of hypotheses and prodigal of facts.*

*Cincinnati, December*, 1834.

---

* The Ethiopians are a people between the extremes of barbarism and civilization. Their garments are of cotton, though those of a more opulent kind are of silk. * * * Unprovided with salt at home, they purchase it from abroad for its weight in gold.—*Buffon. page* 161.

In Norway and Lapland the Scotch Fir-tree attains to a height of 60 feet in latitude 70°; and at *Tornea*, at the head of the Gulf of Bothnia, in latitude 66° the Birches are described by Von Buch as "*magnificent*." * * * In Norway, *Barley* sometimes ripens in favourable aspects, under the 70th parallel of latitude.

The Cape of Good Hope just falls within the latitude adapted to the grape; and a considerable quantity of wine is annually exported from that settlement. It is of very inferior quality to the wines of Europe, and northern Africa, having an unpleasant *earthy* taste, which is said to arise from the clayey nature of the soil.—*Barton's Geography of Plants.*

In New-Holland, a certain species of trees which compose the principal forests, cast or *shed* their extreme bark down to the white *liber* every year; so that though the leaves are ever green, there is a "fall of the bark" answering to the annual "fall of the leaf."—*Mudie's Observations on Nature.*

Separated from the continent by the Straits of Magellan spreads the large but desolate country of Terra del Fuego, and about 300 miles to the north-east of the latter stands the still more barren and inhospitable group of the Falkland Islands.—*Darby.*

The Hottentots seldom live more than forty years, and of this short duration of life, the causes doubtless are, their being so fond of filth, and residing continually in the midst of it; as also their living upon meat which is tainted or corrupted, of which indeed their nourishment chiefly consists.—*Buffon, page* 164.

*The Hyena Dog.*—This dog is a native of Southern Africa, and is a serious nuisance to the frontier settlements at the Cape.—Its ferocity seems to be *untameable.* Mr. Burchell, who first carried it to England, kept one for twelve months, at the end of which period even its feeder did not dare to lay his hand upon it. The Australian dog also is mentioned as exceedingly voracious and fierce.—*Buffon.*

Swine's flesh—it is remarkable, is rejected by the Caffres with abhorrence. The same is the case with the feathered tribe to some extent; none of them keep poultry of any sort; and eggs as an article of food, are altogether contraband. Nay, these scrupulous gentry will have nothing to do with the fish of the sea, which they for the most part regard as company for snakes, and not fit for the food of a gentleman. So that, although these people live almost wholly on or near the coast, the entire line of which abounds with the CHOICEST FISH, they are ignorant of the art of casting a net. * * * * * * The frontier population of the European colony at the Cape is the very *beau ideal* of bastard barbarism. The Caffres and the Dutch boors have always been tugging at each other's throats. * * * * Unfortunately, the English, although only thirty years in possession of the colony, have during that short period outstripped, in their horrible oppression of the natives, even the cold blooded cruelties of the Dutch boors of the last century. No British traveller has denied this, so far as we know, and most of them confirm it in explicit terms. Among these may be named Thompson, Barrow, Pringle, and quite lately, Dr. Philip and the Rev. Mr. Kay.—*N. A. Review for Oct.* 1834.

